# Investigating the role of BDNF in modulation of APP β‐cleavage and BACE1 subcellular distribution

**DOI:** 10.1002/alz70855_105089

**Published:** 2025-12-24

**Authors:** Ryan Douglas Hallam, Natasha K. Fletcher, Gregory Foran, Rebecca EK MacPherson, Aleksandar Necakov

**Affiliations:** ^1^ Brock University, St. Catharines, ON, Canada; ^2^ Centre for Neuroscience ‐ Brock University, St. Catharines, ON, Canada; ^3^ Baylor College of Medicine, Houston, TX, USA; ^4^ Brock University, Department of Health Sciences, St. Catharines, ON, Canada; ^5^ Brock University, Centre for Neurosciences, St. Catharines, ON, Canada

## Abstract

**Background:**

Brain‐derived neurotrophic factor (BDNF) is an important mediator of neuronal function and survival. BDNF levels are elevated in post‐exercise serum extracted from human donors, and we and others have shown that both post‐exercise serum and BDNF alone reduce β‐cleavage of APP within 2 hours of treatment, but the mechanism for this remains unknown. Several other groups have shown that the subcellular location of APP and BACE1 influences APP processing, with α‐cleavage occurring preferentially at the plasma membrane, and β‐cleavage at acidic intracellular compartments, such as endosomes and lysosomes.

**Method:**

Human neuroblastoma cells (SH‐SY5Y) were differentiated for 7 days with 10µM all‐trans retinoic acid. Cells were treated with 75ng/mL BDNF or vehicle control (DMEM/F‐12) for 2 hours as performed previously, and subsequently imaged live via spinning‐disk confocal fluorescence microscopy or collected for Western blotting. Antibodies against sAPPα, sAPPβ, and full‐length APP were used to assess changes in APP cleavage. For live imaging experiments, cells were transfected using Lipofectamine 3000 24 hours prior to treatment with combinations of fluorescently tagged BACE1 (BACE1‐GFP, BACE1‐mScarlet) and subcellular compartment‐specific markers (YFP‐CAAX, mScarlet‐EEA1, LAMP1‐RFP).

**Result:**

Compared to vehicle control, BDNF‐treated cells show a reduction in sAPPβ levels and an increase in the ratio of sAPPα/sAPPβ as measured by Western blot. BDNF treatment also increases colocalization of BACE1 with the plasma membrane marker CAAX and decreases BACE1 colocalization with endosomal marker EEA1. Both phosphorylated BACE1 (Ser498) and BACE1 colocalization with lysosomal marker LAMP1 is unchanged following BDNF treatment. Pretreatment of cells with clathrin‐mediated endocytosis inhibitor PitStop2 (20µM) for 10 minutes prior to BDNF treatment shows increased sAPPα cleavage, but no effect on sAPPβ levels.

**Conclusion:**

Acute treatment with BDNF may decrease β‐cleavage of APP by altering the subcellular distribution of BACE1, increasing recruitment/retention of BACE1 at the plasma membrane and decreasing BACE1 endocytosis. This effect appears to be independent of clathrin‐mediated endocytosis, as PitStop2 treatment increases α‐cleavage of APP but does not reduce β‐cleavage independent of BDNF treatment. Hence, BDNF may reduce production of Aβ by altering BACE1 distribution, decreasing upstream β‐cleavage.

